# *Arabidopsis* HSP90C and SecA1 Have Distinct Client-Binding Modalities to the Thylakoid SEC Client Protein PsbO1

**DOI:** 10.3390/biom16060903

**Published:** 2026-06-18

**Authors:** Adheip Monikantan Nair, Leonardo Tullo, Kenneth Andrei Espinosa, Siu Lun Terrence Tong, Rongmin Zhao

**Affiliations:** 1 Department of Cell & Systems Biology, University of Toronto, Toronto, ON M5S 1A1, Canada; adheip.nair@alumni.utoronto.ca (A.M.N.); leo.tullo@utoronto.ca (L.T.); 2 Department of Biological Sciences, University of Toronto Scarborough, Toronto, ON M1C 1A4, Canada; kennethandrei.espinosa@alumni.utoronto.ca (K.A.E.); terrence.tong@alumni.utoronto.ca (S.L.T.T.)

**Keywords:** HSP90C, SecA1, protein translocation, chloroplast proteostasis, thylakoid membrane, chloroplast stroma

## Abstract

The plastid stroma-localized chaperone HSP90C is essential for maintaining chloroplast proteostasis and facilitating protein translocation. Prior research has established HSP90C’s imperative role in the SEC translocase-dependent transport of the photosystem II subunit PsbO1 and its interaction with the SEC1 translocase motor protein SecA1. However, the exact mechanism of this interaction remains to be explored. In this study, we delineated the interactional mode of HSP90C and SecA1 with the model client protein. Yeast two-hybrid and in vitro ATPase activity analyses with purified proteins revealed PsbO1 may bind to HSP90C at multiple sites, including the DPW motif within the C-terminal extension (CTE) region, suggesting a possible client-loading mechanism unique to plastid orthologs. We also confirmed that glycine-646 is important in mediating substrate interaction, though it conferred a much weaker binding than the CTE region, thereby elucidating a critical role for the amino acid whose mutation resulted in visible plant phenotypes. Our in vitro biochemical assays also demonstrated that the stromal intermediate form of PsbO1 with the thylakoid signal peptide (tSP) significantly enhanced SecA1 ATPase activity, suggesting a preferential binding to the motor protein. On the other hand, the mature domain of the PsbO1, excluding the tSP sequence, inhibited HSP90C ATPase activity. We also observed the HSP90C-PsbO1-SecA1 ternary complex was stabilized by the presence of the client tSP. This work therefore provides new insights into the functional mechanisms of HSP90C and its contribution to chloroplast stromal protein stabilization and thylakoid protein transport.

## 1. Introduction

HSP90C is a plant plastid-localized heat shock protein 90 family protein, and it is essential for plant growth and development, as a knockout mutant in the model flowering plant *Arabidopsis thaliana* results in embryonic lethality [1,2]. Similar to cytosolic HSP90 proteins, which are considered central hubs for cellular protein homeostasis [3], plastid HSP90C also plays a global role in regulating chloroplast differentiation and maturation [4,5,6] and plant growth and development [7]. The function of HSP90C was first characterized by a point mutation line, termed chlorate-resistant 88 (*cr*88) [5], and was more recently evidenced by a pale green mutant (*pga*5-1) at the same amino acid [6]. In *Arabidopsis*, HSP90C interacts with TOC–TIC import components [1], VIPP1 [2] and the thylakoid SEC translocase [4], suggesting its role in protein import, thylakoid membrane biogenesis and protein transport. HSP90C also interacts with the HSP70 protein in green algae to form a foldosome [8]. Particularly, HSP90C physically interacts with the photosystem II protein PsbO1 and the SEC translocase channel protein SecY1 and the motor protein SecA1, indicating the role of HSP90C in guiding thylakoid lumen protein transport through the stroma [4,9,10]. However, how HSP90C and SecA1 specifically interact with the intermediate form of their clients within the stroma remains poorly understood.

The SEC translocon or translocase is a highly conserved protein translocation system essential for maintaining cellular homeostasis and ensuring the proper localization and folding of secretory and membrane proteins [11,12,13,14]. In bacteria, post-translational export relies on SecB and SecA to deliver unfolded pre-proteins bearing N-terminal signal sequences to the SecYEG channel, where ATP-driven SecA cycles push the chain through the SecYEG channel pore [15,16]. In eukaryotes, the Sec61 channel works with the ribosome to co-translationally transport nascent chains to the ER lumen [17]. Chloroplasts in green plants contain two paralogous systems, the SEC1, located in the thylakoid membrane, and SEC2, found in the inner envelope membrane, both crucial for the import of lumenal proteins and the insertion of membrane proteins [18,19]. Given chloroplasts’ prokaryotic ancestry, a mechanism similar to that of the bacterial SecYEG translocase was expected for the plastid SEC translocase, though it lacks the SecG subunit.

In the bacterial post-translational pathway, nascent secretory proteins bearing hydrophobic signal sequences are bound first by trigger factors and then transferred to the SecB chaperone, which maintains them in an unfolded state and delivers them to SecA [20,21,22]. SecB binds signal sequence precursors with ~100-fold higher affinity, stabilizes the client, and hands it directly to SecA, whose ATP-driven cycles feed the polypeptide through SecYEG [16,23,24,25]. Thus, efficient SEC-dependent export universally depends on a cognate N-terminal signal sequence. Despite plastid systems lacking a SecB orthologue, stromal heat shock proteins (HSPs) have been implicated as functional substitutes. For instance, stromal chaperonin Cpn60 transfers an ‘insertion-competent’ form of the luminal peptidase Plsp1 to SecA1 [26]. HSP90C interacts with both the soluble protein PsbO1 and the thylakoid membrane protein LHCB2 [10], and it may be another heat shock protein that plays an even broader role in stabilizing and aiding in protein transport to the thylakoid.

Like all HSP90 family proteins, HSP90C contains an N-terminal ATP-binding domain (NTD), middle domain (MD), and dimeric C-terminal domain (CTD) and harbors a C-terminal extension region (CTE) with a uniquely conserved Asp-Pro-Trp (DPW) motif found only in green lineage HSP90Cs [27]. HSP90s promote client maturation through nucleotide-regulated conformational cycles coordinated by cochaperones in the cytoplasm [28,29,30]. For organellar or prokaryotic HSP90 proteins, the information on co-chaperone-aided protein folding is very limited, and the HSP90C CTE has been proposed to bind and intrinsically regulate the client binding and functional cycle [27], but how it may be involved in the thylakoid lumen protein binding and then the SEC translocase-dependent protein transport is unclear.

In this study, we used biochemical assays to further elucidate the mechanism of the HSP90C-mediated SEC1 translocation pathway. Building on this ternary complex hypothesis, we conducted ATPase assays by titrating varying amounts of PsbO1 proteins containing the tSP and those lacking it. We demonstrated that the tSP preferentially binds SecA1, whereas HSP90C preferentially binds the mature domain of PsbO1, suggesting distinct binding modalities. Furthermore, the conserved DPW motif is crucial in the chaperone’s ATPase-dependent function and client interactions. Therefore, our work adds key details to HSP90C’s interaction with client proteins and the SEC1 translocation process, which helps us postulate in vivo scenarios.

## 2. Materials and Methods

### 2.1. Plasmid Construction

Constructs for His6-tagged mature HSP90C (HSP90C), 30 amino-acid CTE-truncated His6-tagged mature HSP90C (HSP90C^ΔC30^) and His6-tagged mature PsbO1^T200A^ (*m*PsbO1^T200A^) in pProEX HTb were generated as described previously [10,27]. His6-tagged *m*SecA1 expressed in pET28a was a kind gift from Dr. Donna E. Fernandez (University of Wisconsin–Madison). To express and purify *i*PsbO1 and *m*PsbO1, plasmids encoding *i*PsbO1–GFP and *m*PsbO1–GFP based on pET22b [10] were cleaved by NcoI and the GFP coding sequence was removed after self-ligation. All constructs were transformed into *E. coli* BL21 (DE3)-pRIL (Stratagene) under ampicillin or kanamycin selection for protein expression. To express and purify mutant HSP90C proteins, site-directed mutagenesis was performed on pProExhtb-mHSP90C (mature form, lacking the chloroplast targeting peptide) to introduce mutations within the C-terminal extension with specific primers. The primers used for the site-directed mutagenesis and the final bait plasmids were indicated in Supplementary Tables S1 and S2, respectively.

### 2.2. Protein Expression and Purification in E. coli Under Native Conditions

BL21(DE3) cells harboring pProEX-HTb–HSP90C^ΔC30/ΔDPW/ΔEPSE/G646R^ and pET28a–SecA1 were all transformed into *E. coli* BL21 (DE3)-pRIL (Stratagene) carrying ampicillin or kanamycin resistance. For protein expression, each construct was grown in LB medium containing 100 μg/mL ampicillin or 50 μg/mL kanamycin, respectively, induced with 0.4 mM IPTG at OD600 ~2, and incubated at 27 °C for ~16 h. Pelleted cells were resuspended in lysis buffer (25 mM Tris-HCl pH 7.5, 100 mM KCl, 10% glycerol, 1 mM DTT, 5 mg/mL lysozyme), mechanically disrupted by sonication (Branson, Danbury, CT, USA), and cleared by centrifugation at 10,000× *g* for 30 min. The supernatant was applied to Ni-NTA resin (QIAGEN, Venlo, The Netherlands), pre-equilibrated in wash buffer (25 mM Tris-HCl pH 7.5, 100 mM KCl, 10% glycerol), then washed with the same wash buffer, and eluted in elution buffer (25 mM Tris-HCl pH 7.5, 150 mM NaCl, 10% glycerol, 150 mM imidazole, 0.5 mM DTT). The eluate was subjected to size-exclusion chromatography on the Superdex 200 10/300 GL column fitted in the fast protein liquid chromatography (FPLC) ÄKTApurifier 10 (Cytiva, Vancouver, BC, Canada) system. Bradford assay was used to quantify pooled fractions containing HSP90C dimers or SecA1 monomers, and the representative SDS-PAGE gels for the purified HSP90C proteins after separation by size exclusion chromatography were shown in Supplementary Figure S1c, while Supplementary Figure S1d shows the SDS-PAGE gel of final purified proteins used for ATPase activity assays etc. The monomeric form of SecA1 has been previously confirmed [9].

### 2.3. Protein Expression and Purification in E. coli Under Denaturing Conditions

BL21(DE3) cells harboring the pET22b-PsbO1 constructs were grown and induced as above, then centrifuged at 10,000× *g* for 30 min at 4 °C. Pellets were resuspended in 8 M urea lysis buffer (100 mM NaH_2_PO_4_, 10 mM Tris-HCl pH 8.0) for two hours on ice, cleared by centrifugation, and loaded onto Ni-NTA resin equilibrated in denaturing wash buffer (10 mM Tris-HCl pH 8.0, 8 M urea, 100 mM NaH_2_PO_4_). After washing with 20 mM imidazole added in the same denaturing wash buffer, proteins were eluted with denaturing elution buffer (25 mM Tris-HCl pH 7.5, 8 M urea, 150 mM NaCl, 10% glycerol, 150 mM imidazole, 0.5 mM DTT), then dialyzed overnight at 4 °C against renaturation buffer (25 mM Tris-HCl pH 7.5, 150 mM NaCl, 10% glycerol, 0.5 mM DTT). The partially refolded samples were subjected to size-exclusion chromatography on a Superdex 200 10/300 GL column, and fractions corresponding to monomeric PsbO1 were pooled, concentrated, quantified by Bradford assay, and stored at −80 °C. Representative SDS-PAGE images of the size exclusion chromatography fractionations of the three different PsbO1 isoforms were shown in Supplementary Figure S1b.

### 2.4. Steady-State ATP Hydrolysis Activity Assay

ATP hydrolysis activity was measured using the NADH-coupled method as previously described [31]. Purified HSP90C, SecA1 or PsbO1 proteins were mixed in equimolar compositions (1.41 μM) in the assay reaction mixture consisting of 25 HEPES (pH 7.5), 5 mM MgCl_2_, 500 mM KCl, 0.03% Tween 20, 10% glycerol, 200 μM NADH, 3 mM phosphoenol pyruvate, 15.7 U of pyruvate kinase (MilliporeSigma, Oakville, ON, Canada), and 24.5 U of lactate dehydrogenase (Sigma, St. Louis, MO, USA). The extinction of NADH in the reaction mixture was mapped over time using the Synergy 4 microplate reader (BioTek, Winooski, VT, USA). Wherever needed, 25 μM radicicol and 20 mM sodium azide were used to specifically inhibit the HSP90C and SecA1 ATPase activity, respectively. The turnover rate (kcat) was analyzed using GraphPad Prism.

Client-protein titration ATPase experiments were conducted with 1.41 μM of HSP90C or SecA1 and increasing concentration of indicated PsbO1 constructs (0.05–3 μM). Fit lines concerning client-induced ATPase stimulation observed with SecA1 were calculated according to the classical Michaelis-Menten model using the following equation (Y = Vmax × X/(Km + X)), where Vmax and KM were both determined using GraphPad Prism (Version 9.5.1). Fit lines concerning client-induced ATPase inhibition observed with HSP90C were calculated using the following equation Y = (Bmax × Xn)/(Kappn + Xn) + X0 using GraphPad Prism (Version 9.5.1). All curve fits had R2 values greater than 0.9.

### 2.5. Yeast Two-Hybrid

The pEG202–HSP90CMC bait construct and the pJG4-5 *m*PsbO1^T200A^ prey vector, derived from an *Arabidopsis* cDNA library, were described previously [10,27]. Briefly, HSP90CMC (residues 320–780 of *Arabidopsis* HSP90C, spanning the middle domain through the C-terminal extension) was PCR-amplified and cloned into pEG202 at EcoRI/NcoI sites. The pJG4-5 *m*PsbO1^T200A^ prey vector was derived from an *Arabidopsis* cDNA library. To generate HSP90CMC mutant constructs, the HSP90CMC fragment from pEG202–HSP90CMC was cloned into the pProExhtb vector between EcoRI and NcoI sites, and site-directed mutagenesis of the DPW and EPSE motifs or G646 was employed using standard Pfu-based PCR and DpnI digestion to remove parental templates and subsequent transformations, with correct mutations verified by Sanger sequencing. After mutation, the HSP90CMC fragments were cloned back into pEG202 using the EcoRI and NcoI sites. The primers used for the site-directed mutagenesis and the final bait plasmids were indicated in Supplementary Tables S1 and S2, respectively. Yeast two-hybrid assays used Saccharomyces cerevisiae EGY48 carrying the p8opLacZ, transformed with bait (HSP90CMC variants) and prey (*m*PsbO1^T200A^) plasmids, and plated on selective media supplemented with galactose for assessing interaction strength. Protein expression in yeast was verified by immunoblotting with anti-HA or anti-LexA antibodies, confirming the presence and integrity of the expressed proteins and the original blots were shown in Supplementary Figure S2.

### 2.6. Structural Models Prediction with AlphaFold3

The protein structures of dimeric HSP90C, monomeric SecA1 and PsbO1 were initially generated using AlphaFold3 [32] from the protein sequences listed in Table S3. The reliability of the predicted structures was examined and compared with the experimentally resolved structures, and the associated predicted Local Distance Difference Test scores for HSP90C, SecA1 and the different isoforms of PsbO1 are shown in Supplementary Figures S3, S4 and S5, respectively, together with experimentally solved structures as references.

### 2.7. Molecular Dynamics Simulations

Molecular dynamics simulations were performed on the SciNet HPC Consortium supercomputer (https://scinethpc.ca/, accessed on 1 November 2024) using GROMACS (2024.1) with the CHARMM27 all-atom force field, optimized for protein simulations and compatible with the TIP3P water model [33,34]. Specifically, simulations of the SecA1 and PsbO1 proteins were conducted with CHARMM27 for single-protein analysis and CHARMM36 for multi-chain analyses [35]. The AlphaFold generated structures were subsequently prepared by adding missing hydrogen atoms and adjusting ionization states to a pH of 7. The system was solvated in a cubic box containing TIP3P water molecules, with a 2.5 nm buffer. Counterions (K^+^ and Cl^−^) were added to neutralize the system and achieve a physiological concentration of 100 mM KCl. Energy minimization was performed using the steepest descent algorithm until the maximum force reached 1000 kJ mol^−1^ nm^−1^. This process was followed by a two-step equilibration: the first step involved a canonical ensemble (constant number of particles, volume, and temperature) (NVT) at 300 K using a 1 ns velocity-rescale thermostat (τ = 0.1 ps) with a separate temperature coupling groups for protein and non-protein atoms, followed by a 1 ns isothermal-isobaric ensemble (constant number of particles, pressure, and temperature) (NPT) using the Parrinello-Rahman barostat (τ = 2.0 ps) to ensure pressure stabilization at 1 bar, compressibility of 4.5 × 10^−5^ bar^−1,^ and reference coordinate scaling. The production molecular dynamics runs were extended to 250 ns for PsbO1 and 100 ns for SecA1, with a time step of 2 fs under periodic boundary conditions in all directions, and neighbor lists updated every 10 steps (20 fs) using the Verlet cutoff scheme [36]. The particle-mesh Ewald method was used for long-range electrostatic interactions [37,38] with a 1.2 nm cutoff and a 0.12 nm grid spacing, while van der Waals interactions were treated with a 1.2 nm cutoff, with long-range dispersion corrections applied to energy and pressure. All bond lengths were constrained using the LINCS algorithm [39]. Trajectories were written every 5 ps in a compressed format, while energy, temperature, pressure, and log outputs were recorded every 2 ps. Structural stability and residue-specific flexibility were validated using root-mean-square deviation (RMSD) & root mean square fluctuation (RMSF) profiles over the simulation timeframe.

### 2.8. Antibodies

Monoclonal mouse anti-Lex A antibody was purchased from Santa-Cruz Biotechnology (College Park, MD, USA), and monoclonal anti-HA was purchased from Invitrogen (part of Thermo Fisher Scientific, Ottawa, ON, Canada). Rabbit anti-HA and mouse anti-LexA antibodies are used to detect prey and bait vectors.

## 3. Results

### 3.1. HSP90C DPW Motif Is Required for Efficient Client Interaction

**Figure 1:**
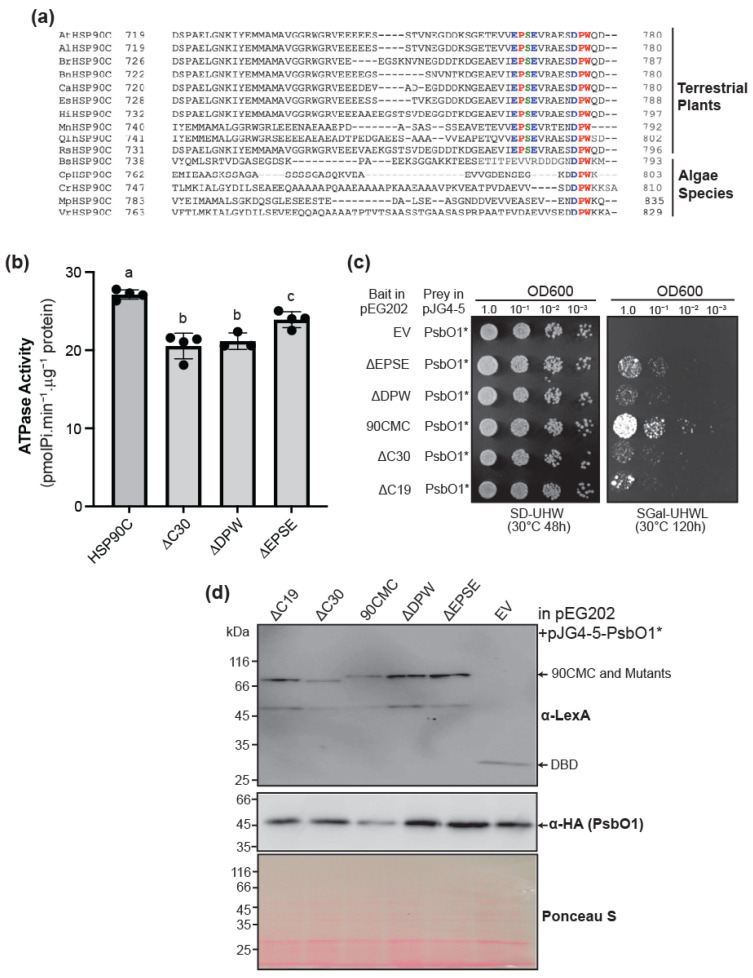
The effect of conserved motifs within the CTE on HSP90C’s ATPase activity and client interaction. (**a**) Clustal Omega alignment of HSP90C C-terminal segments from representative land plant and algal species. Highly conserved DPW and EPSE motifs are highlighted, and their preservation across angiosperms, bryophytes, and green algae is compared. The charged, polar and hydrophobic aminos acids are indicated in blue, green and red, respectively. (**b**) ATPase hydrolysis of His6-HSP90C, HSP90C^ΔC30^, HSP90C^ΔDPW^, and HSP90C^ΔEPSE^ at 5 mM ATP. Bar plots represent mean ± SD from four technical replicates, with significance assessed via one-way ANOVA, and letters above the error bars indicate statistically significant differences between the two groups (*p* < 0.05). (**c**) Yeast two-hybrid assay evaluating the interaction between PsbO1^T200A^ (PsbO1*) and 90CMC truncation mutant constructs. Yeast cells (OD600 = 1) were serially diluted 10-fold and spotted onto SD-UWH (control) and SGal-UWHL (interaction) media to visualize binding. Empty vectors with bait or prey proteins were used as negative controls. (**d**) Immunoblots verifying expression of yeast two-hybrid constructs. Yeast strains harboring bait and prey plasmids were probed with anti-LexA and anti-HA antibodies to confirm comparable expression levels of the respective proteins. See original Western blot images in Supplementary Figure S2.

Previous studies indicated that a DPW motif within the HSP90C CTE is highly conserved across green lineages (Viridaeplantae) [27], suggesting a critical functional role in HSP90C. Comparative analysis of HSP90C orthologs, particularly from terrestrial plant species, identified a segment spanning EPSE that may also be pivotal for HSP90C’s functionality (Figure 1a). To understand whether these conserved motifs are sufficient to mediate the chaperone’s ATPase activity, we used site-directed mutagenesis and deleted the respective DPW or the EPSE motifs and re-examined the ATPase activity of the purified mutant proteins. As controls, we examined the ATPase activity of the full-length HSP90C and of the HSP90C^ΔC30^, confirming the CTE’s importance in enhancing the ATP hydrolysis (Figure 1b) as previously reported [27]. Surprisingly, deleting the EPSE motif (HSP90C^ΔEPSE^) caused an ~11.7–11.9% reduction compared to full-length HSP90C. By contrast, the highly conserved DPW motif had an even more significant effect. The deletion of the DPW motif (HSP90C^ΔDPW^) caused a ~17.3% decrease (Figure 1b). The DPW motif deletion significantly impaired ATPase activity relative to the HSP90C^ΔEPSE^ mutant and the full-length protein, indistinguishable from that of HSP90C^ΔC30^. This demonstrates that both DPW and EPSE motifs contribute to optimal ATP hydrolysis in HSP90C, with DPW deletion exerting the most pronounced effect.

To determine whether these conserved motifs are sufficient to mediate interaction with its client, we verified the interaction with the mutant *m*PsbO1^T200A^ by yeast two-hybrid as previously reported [10]. Interestingly, HSP90C^ΔEPSE^ also produced a modest decrease in yeast growth, akin to the previously characterized 90CMC^Δ19^ truncation (Figure 1c) [27]. The HSP90C^ΔDPW^ mutant showed a markedly more significant impairment in HSP90C-PsbO1 interaction, with noticeably reduced colony growth compared to HSP90C^ΔEPSE^, although the expressions of the bait and prey proteins are comparable in the test stains (Figure 1d). Collectively, this suggests that the DPW motif constitutes a critical binding site mediating client interaction, and the interaction may be also strengthened by the upstream amino acids.

### 3.2. HSP90C Engages with the PsbO1 Client Protein Through More Than One Binding Site

**Figure 2:**
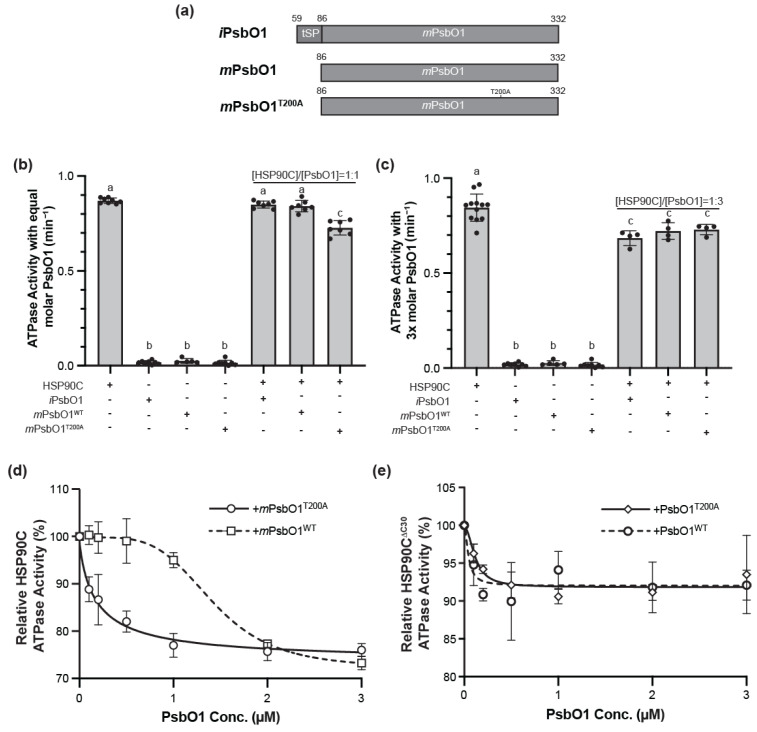
Effect of PsbO1 proteins on HSP90C and HSP90C^ΔC30^ ATPase activity. (**a**) A schematic representation of *i*PsbO1, *m*PsbO1 and *m*PsbO1T200A proteins used in this study. (**b**,**c**) The ATPase activity for HSP90C was measured in the absence and presence of equal (**b**) or excess molar mixes (**c**) of either *i*PsbO1, *m*PsbO1 or *m*PsbO1^T200A^. (**d**,**e**) The ATPase activity of HSP90C (**d**) and HSP90C^ΔC30^ (**e**) with increasing molar concentrations of *i*PsbO1, *m*PsbO1 or *m*PsbO1^T200A^. ANOVA was performed, and letters above the error bars indicate statistically significant differences between the two groups (*p* < 0.05).

To better understand the client binding dynamics between HSP90C and PsbO1, we constructed three versions of the PsbO1 protein: the wild-type mature form of PsbO1 (*m*PsbO1), the intermediate form of PsbO1 (*i*PsbO1) that contains the tSP, and the mutant mature form of PsbO1 (*m*PsbO1^T200A^) (Figure 2a). The purified monomeric forms of these PsbO1 proteins were used to test their interaction and influence on HSP90C ATPase activity. It was surprisingly observed that neither *m*PsbO1 nor *i*PsbO1 produced any significant stimulatory or inhibitory effect on the ATPase activity of HSP90C when mixed with equal molar amounts (Figure 2b). Agreeing with previous reports, the mutant variant *m*PsbO1^T200A^ exhibited an approximate 14% inhibitory effect on the basal ATPase activity of HSP90C. We subsequently applied excessive amounts of *m*PsbO1 and *i*PsbO1, using three times the molar amount of HSP90C, and observed a decrease in ATPase activity, reaching an inhibitory level comparable to that observed with *m*PsbO1^T200A^ (Figure 2c). We propose that this could be because the wild-type PsbO1 interacts less effectively with HSP90C than the mutant *m*PsbO1^T200A^, as previously noted [10]. To better understand the binding dynamics, we tracked the effects of mPsbO1 and the mutant *m*PsbO1^T200A^ at increasing concentrations. We observed that *m*PsbO1^T200A^ exhibited an inhibitory effect mimicking a hyperbolic model, while mPsbO1 displayed a more complicated sigmoidal inhibitory model (Figure 2d). The maximal inhibitory effects were comparable for both proteins, exhibiting approximately 25% inhibition when three times the molar equivalents of PsbO1 proteins were incorporated into the reaction mixture.

HSP90C’s CTE region is required for optimal client binding (Figure 1). To understand the impact of the CTE-PsbO1 interaction on the HSP90C ATPase activity, we re-examined the effect of the wild-type *m*PsbO1 and *m*PsbO1^T200A^ on the CTE-truncated HSP90C^ΔC30^ protein. Interestingly, both proteins displayed only a minor inhibitory effect on HSP90C^ΔC30^ activity, and there was no visible difference between the two proteins; in particular, the sigmoidal response was abolished in the *m*PsbO1 treatment (Figure 2e). The CTE of HSP90C likely provides a primary and high-affinity binding site for the PsbO1 protein. In contrast, the HSP90C middle or C-terminal domain may provide a common, but weaker binding site for all client proteins, contingent on its conformational cycle.

### 3.3. HSP90C C-Terminal G646 Loop Interacts with PsbO1

**Figure 3:**
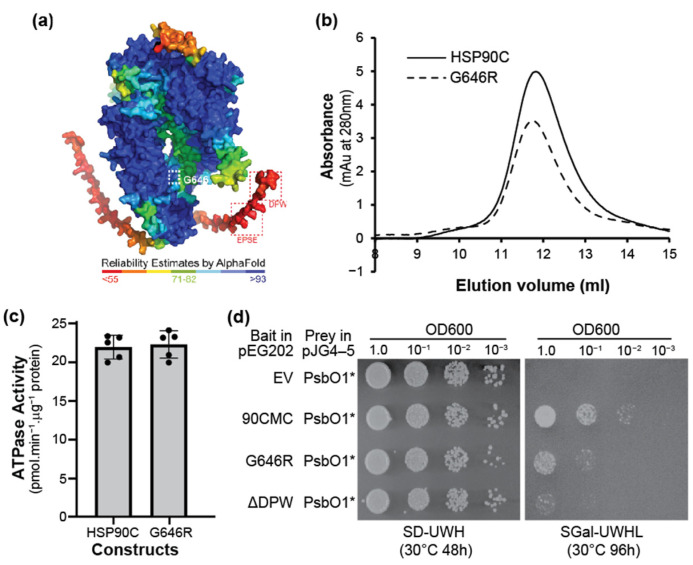
HSP90C G646 region is required for PsbO1 interaction. (**a**) Surface model of predicted HSP90C dimer structure to highlight the FG646W loop (with G646 in the dashed line box) on the exposed external site from the dimer lumen region. The C-terminal DPW and EPSE motifs are also indicated in boxes. The colors depict the predicted local distance difference Test (pLDDT) scores (**b**) Size exclusion chromatography profile of wildtype mature HSP90C and the G646R mutant protein. (**c**) The ATPase activity of HSP90C and HSP90C^G646R^ proteins. (**d**) Yeast two-hybrid assay evaluating the interaction between PsbO1^T200A^ (PsbO1*) and 90CMCG646R mutant constructs. Yeast cells (OD600 = 1) were serially diluted 10-fold and spotted onto SD-UWH (control) and SGal-UWHL (interaction) media to visualize binding. Empty vectors with bait and the wildtype HSP90CMC were used as negative and positive controls, respectively.

The originally identified cr88 mutant contains a G646R point mutation [5], and a recent study reported that another point mutation, G646E, at the same amino acid also resulted in pale green leaves and was designated pga5-1 [6]. The region around G646 in HSP90C is predicted to adopt a loop structure located at the outmost surface of the large luminal substrate binding region from the closed dimer structure (Figure 3a), which is also conserved in other HSP90 family members [40]. We speculated that G646 and the loop may be an important site to regulate the protein function and then expressed and purified the G646R mutant protein from *E. coli*. This mutant protein appeared as a dimer, similar to the wild-type protein (Figure 3b), and was eluted at about 11.80 mL in our Superdex 200 size-exclusion column, as previously observed [27]. The ATPase activity of the mutant form is also similar to the wildtype protein (Figure 3c). However, our yeast two-hybrid analysis indicated that G646R mutation dramatically reduced its interaction with the substrate PsbO1 (Figure 3d). This suggests that the G646 Loop may be part of the critical substrate-binding site, at least for the photosynthetic protein PsbO1.

### 3.4. PsbO1 Thylakoid Signal Peptide Stimulates SecA1 ATPase Activity

**Figure 4:**
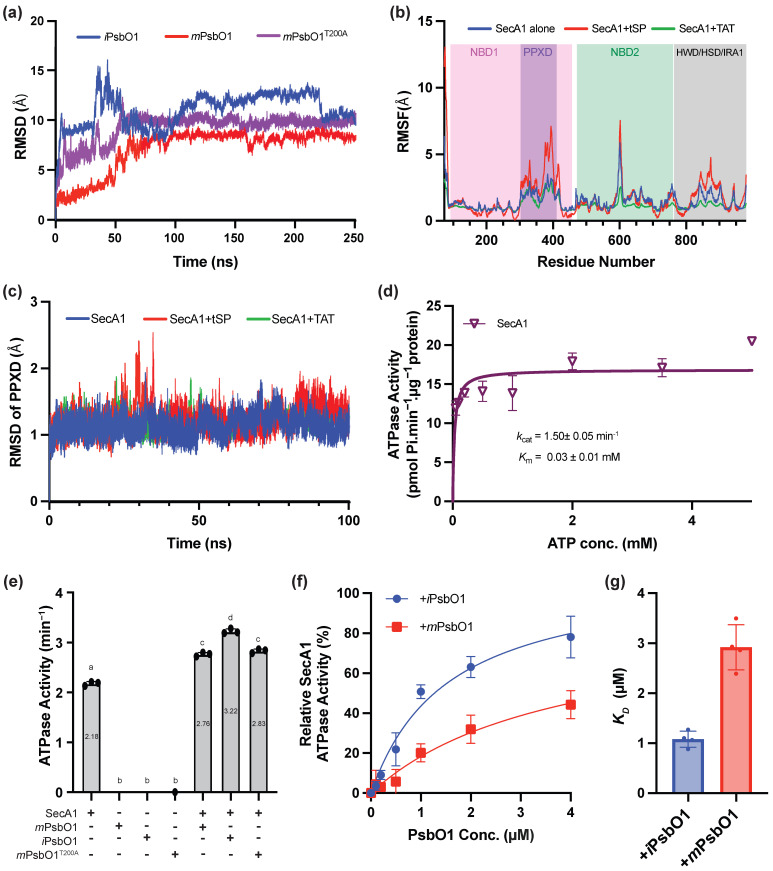
The effect of the tSP on SecA1 ATPase activity. (**a**) The RMSD profile of the entire protein backbone Cα atoms was computed for *i*PsbO1 (blue), *m*PsbO1 (red) and *m*PsbO1^T200A^ (purple). The RMSD profiles were obtained from a simulation in a 100 mM KCl environment for a duration of 250 ns. (**b**) The RMSF profiles of the protein backbone Cα atoms were computed for SecA1 alone (blue), SecA1 + tSP (red) and SecA1 + TAT (green) treatments. The structural domains of SecA1 are as follows: NBD1 in pink, PPXD in purple, NBD2 in green, and a composite domain consisting of the HWD, HSD, and IRA1 represented collectively in grey, with the CTT shown in red. The x-axis begins at residue number 73, corresponding to S73 on the mature SecA1 protein sequence. (**c**) The RMSD profile for the protein backbone Cα atoms was computed for SecA1 alone (blue), SecA1 + tSP (red) and SecA1 + TAT (green) treatments, focusing specifically on the Cα atoms within the PPXD of SecA1. The RMSD profiles were simulated in a 100 mM KCl (pH 7.0) environment for a duration of 100 ns. (**d**) ATP hydrolysis rates of His6-tagged SecA1 construct across different ATP concentrations. Error bars indicate standard deviations from at least five replicates per concentration. Michaelis constant (*K*_M_) and turnover rate (*k*_cat_) for SecA1 are shown. (**e**) The ATPase activity of SecA1 was measured in the absence or presence of equimolar *m*PsbO1, *i*PsbO1 and *m*PsbO1^T200A^. ANOVA was performed, and letters above the error bars indicate statistically significant differences between the two groups (*p* < 0.05). (**f**) The relative ATPase activity of SecA1 was evaluated at increasing molar concentrations of the wild-type mature and intermediate form PsbO1, the *m*PsbO1 and *i*PsbO1, respectively. (**g**) *K*_D_ values for *m*PsbO1 and *i*PsbO1 treatments are calculated using a classical Michaelis-Menten model, with data expressed as mean ± SD from four independent replicates.

To elucidate differences in the effect of mutant client protein on HSP90C ATPase activity, we examined the structural integrity of the PsbO1 variants over 250 ns using MD trajectories and observed distinct stability hierarchies. *m*PsbO1^T200A^, containing the point mutation, yielded an average RMSD of ~9.9 Å (Figure 4a). In contrast, the mPsbO1 structure stabilizes with an average RMSD of ~8.3 Å, suggesting that the T200A substitution collates the structure in a relatively higher energy and dynamic ensemble compared to its wild-type counterpart. *i*PsbO1, which retains the tSP, shows the most significant RMSD amplitude (~8–15 Å) (Figure 4a) and persistent fluctuations reflecting the intrinsic disorder and frequent repositioning of the tSP sequence. Together, these trends suggest that the T200A mutation yields a slightly more destabilized intermediate that may confer stronger affinity for HSP90C.

Previous works have distinguished cpSecA1 and *E. coli* SecA to bind their cognate signal peptides preferentially. tSP from pea plastocyanin and PsbO1 stimulate cpSecA1 by ~30%, whereas bacterial SEC signal peptides from alkaline phosphatase (AP) and β-lactamase (β-lac), as well as the chloroplast TAT signal peptides from SufI and the 23-kDa OEC subunit (OE23), have minimal effect [41]. However, the dynamics of this preferential binding are much less understood. To elucidate the atomistic mechanisms underlying binding events and dynamics between SecA1 and tSP, MD simulations were conducted to monitor Cα dynamics within the protein backbone in the absence and presence of an isolated tSP sequence from PsbO1 and a twin-arginine translocation (TAT) sequence from PsbQ1. The RMSD and RMSF trajectory analyses from a 100 ns simulation conducted in a 100 mM KCl (pH 7) environment lacking ATP revealed that the tSP sequence induces movement in backbone residues primarily associated with the PPXD and HWD/HSD/IRA1 domains, suggesting a potential interaction (Figure 4b). The TAT sequence, which has been demonstrated not to interact with SecA1 [41], expectedly did not exhibit any movement in the backbone residues of SecA1 (Figure 4b). The interactional mode between SecA1 and the tSP mimics to that of *E. coli* SecA’s interaction with bacterial maltoporin protein signal peptide (PDB: 2VDA) attained through NMR analysis [42]. Given that the PPXD was the most significantly impacted domain, the RMSD of the Cα atoms from the PPXD was assessed for its general dynamics over time in both the absence and presence of the tSP and the TAT sequences (Figure 4c). Only the presence of the tSP showed a domain specific generalized deviation of SecA1’s PPXD domain by ~0.5 Å from the 80 ns mark onwards (Figure 4c). Collectively, the in silico predictions suggest that the tSP is important in initiating the binding process.

To validate the simulation data that SEC targeting tSP induces more dynamic movement of the SecA1 domains, we expressed, purified and measured the ATPase activity of the SecA1 protein in the absence and presence of different versions of the PsbO1 proteins. It turns out that SecA1 had an estimated binding affinity (*K*_M_) of 0.03 ± 0.01 mM to ATP molecules with a turnover rate (*k*_cat_) of 1.50 ± 0.05 min^−1^ (Figure 4d). We then co-incubated SecA1 with equimolar amounts of PsbO1 protein and observed that iPsbO1 exhibited a notable enhancement in SecA1 ATPase activity, with an approximate increase of 48% relative to its basal ATPase activity (Figure 4e). This finding suggests that the tSP substantially strengthens the interaction with SecA1. Conversely, mature constructs of PsbO1, including the wild-type *m*PsbO1 and the *m*PsbO1^T200A^ variant, stimulated SecA1 ATP activity by approximately 27–30% (Figure 4e). We observed no difference between the stimulation of SecA1’s ATPase activity induced by the *m*PsbO1 and the *m*PsbO1^T200A^ constructs, implying that the PsbO1’s mature domain may not favorably bias the interaction to SecA1 in comparison to the tSP.

To further investigate the binding dynamics between SecA1 and its SEC client proteins, ATPase assays were performed by including client proteins at varying micromolar concentrations. Given the marked differences observed between the intermediate and mature forms of PsbO1 in their ability to stimulate SecA1 ATPase activity, measurements were conducted using both *i*PsbO1 and *m*PsbO1. The assays demonstrated that both *i*PsbO1 and *m*PsbO1 significantly enhance SecA1 activity, with a pronounced effect as client protein concentration increases (Figure 4f). *i*PsbO1 substantially outperformed *m*PsbO1 in stimulating SecA1 activity when administered in equivalent molar amounts. The data yielded *k*_D_ values of 1.08 µM and 2.92 µM for *i*PsbO1 and *m*PsbO1, respectively, for the SecA1-PsbO1 interactions (Figure 4g). These results underscore the enhanced binding affinity of the tSP for SecA1, highlighting the critical, conserved role of the tSP in SecA1 functionality.

### 3.5. PsbO1 Thylakoid Targeting Peptide Potentiates SecA1-HSP90C Interaction

**Figure 5:**
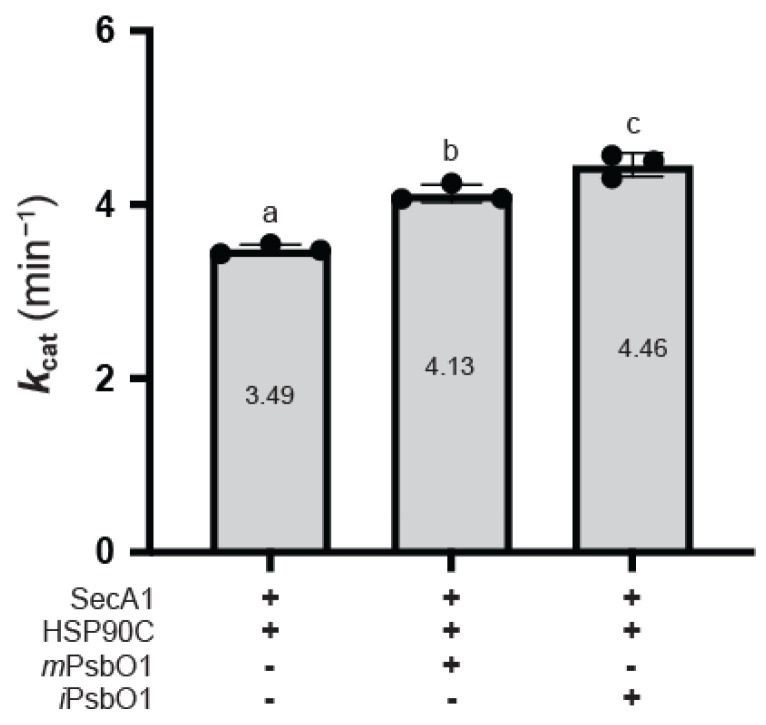
The effect of the tSP on the combined HSP90C and SecA1 activity. The ATPase activity (*k*_cat_) values for the equal molar mixtures SecA1 and HSP90C in the absence or presence of equal molar PsbO1 constructs. Error bars denote the SD from three trials. ANOVA was performed, and letters above the error bars indicate statistically significant differences between the two groups (*p* < 0.05).

**Figure 6:**
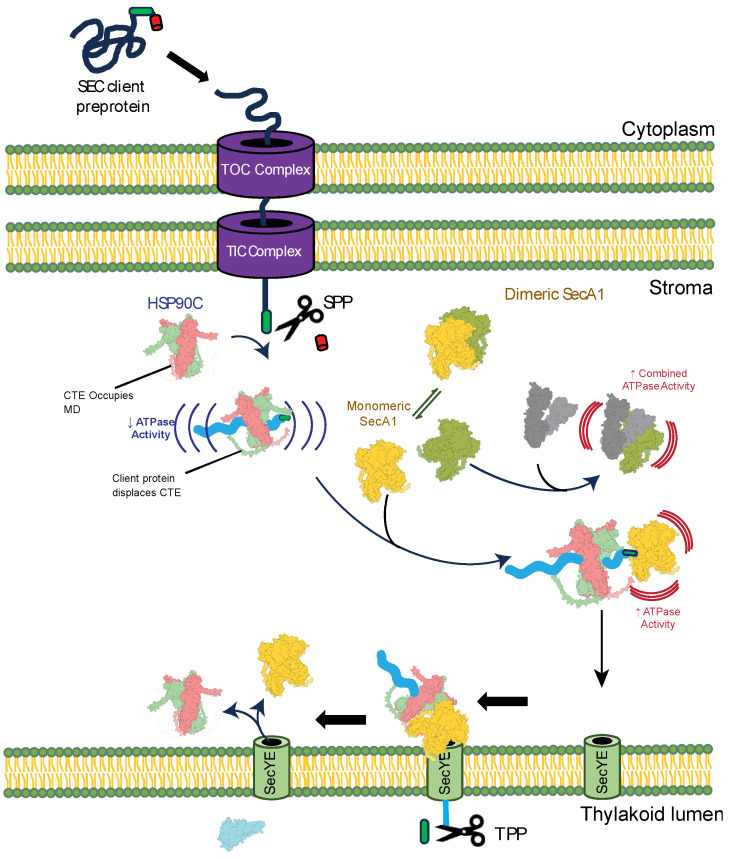
The proposed HSP90C and SecA1 interaction mechanism during preprotein translocation in the plastid thylakoid SEC1 system. Dimeric HSP90C (pink-green) stabilizes the SEC client protein (cyan) in the stroma, recruiting monomeric SecA1 (yellow) via its tSP (green) and forming a complex targeting the SecYE channel (light green). ATPase activity inhibition and stimulation are represented by gapped blue ripples and bunched red ripples, respectively. The model emphasizes the interaction between HSP90C, SecA1, SEC client proteins, and the thylakoid SecYE channel, excluding co-chaperones and other auxiliary plastid components. Direct interaction between HSP90C (grey) and monomeric SecA1 (lime green) is also proposed.

PsbO1 has previously been implicated in the formation of the SecA1-PsbO1-HSP90C ternary complex [9]. To assess whether tSP enhances ternary complex formation, we measured overall ATP hydrolysis activity by combining HSP90C and SecA1 in the reaction mixture, supplemented with varying concentrations of PsbO1 constructs. When *i*PsbO1 was co-incubated with both SecA1 and HSP90C, the resultant combined ATP turnover rate exhibited a 28% increase compared to the combined turnover rate of HSP90C and SecA1 alone, representing an approximately 10% greater enhancement than observed with *m*PsbO1 (Figure 5). This finding indicates that the presence of the tSP on the SEC client protein potentiates the interaction between SecA1 and HSP90C. Overall, our findings substantiate that SecA1 and HSP90C exhibit distinct binding modalities concerning their interactions with the SEC client protein. These results therefore provide evidence for the formation of a ternary complex involving HSP90C, SecA1, and the client protein, with SecA1 specifically binding to the N-terminal tSP of PsbO1 and HSP90C binding further downstream in the PsbO1 sequence. We hypothesize that this distinct binding mode protects PsbO1 in the plastid stroma and ensures its appropriate targeting to the thylakoid SEC translocase (Figure 6).

## 4. Discussion

HSP90C is a plastid stroma-localized molecular chaperone, and its essential role has been evidenced by analyses of point-mutant mutants [6], knockdown [7], and knockout lines [2]. Direct physical interactions have been established between HSP90C and the TOC/TIC translocon [1] as well as the thylakoid SEC translocase subunits [4]. Particularly, the photosystem II subunit PsbO1 and LHCB2 were confirmed to interact with the HSP90C, being considered as favorable candidate clients of the molecular chaperone [10]. In this study, we used PsbO1 as a model client and identified short client-binding motifs within the HSP90C CTE region and in the region around the G646 loop. PsbO1 binds the CTE region with a high affinity to the terminal DPW motif (Figure 1 and Figure 2) and binds the G646 region (Figure 3) region with a relatively low affinity. Additionally, we confirmed that the PsbO1 thylakoid targeting peptide binds and stimulates SecA1, the SEC motor protein, while the mature form of PsbO1 mainly engages its interaction with HSP90C (Figure 4), and the intermediate form iPsbO1 significantly potentiates the ATPase activity when SecA1 and HSP90C are in the same complex (Figure 5). Taken all together, HSP90C has a global function within the plastid. Briefly, HSP90C facilitates plastid protein import via the TOC-TIC complex, stabilizes unstable proteins in the stroma, and then guides substrates to the thylakoid membrane for lumen transport when clients are destined for lumen localization (Figure 6).

### 4.1. Chloroplast HSP90C Utilizes a Distinct Interactional Mode with Its Clients

Our ATPase assays revealed distinct binding dynamics between HSP90C and PsbO1. A mutant form of mature PsbO1, *m*PsbO1^T200A^, exhibited significantly stronger interaction with HSP90C than wild-type *m*PsbO1 [10]. This was confirmed by ATPase activity assays, in which *m*PsbO1^T200A^ induced a pronounced inhibition of HSP90C ATPase activity, indicating greater client-binding potency (Figure 2d). Our ATPase assays also suggest that HSP90C preferentially binds to sequences along the mature domain, as evidenced by the pronounced interaction with *m*PsbO1^T200A^ in comparison to *i*PsbO1 and *m*PsbO1 (Figure 2b). Interestingly, the presence of the tSP on the client protein had a negligible effect on HSP90C binding (Figure 2c).

When comparing HSP90C to its CTE-truncated variant, HSP90C^ΔC30^, neither *m*PsbO1 nor *m*PsbO1^T200A^ displayed any significant difference in their responses (Figure 2e). This suggests that removing the CTE dramatically reduces the chaperone’s ability to bind client proteins effectively. In the presence of the CTE, client binding likely competes with intramolecular interactions involving the CTE, modulating client loading onto the chaperone. Similar client-induced inhibition has been observed in other HSP90 orthologs, such as GR-bound human HSP90 [43]. However, plastid HSP90C appears unique in its use of its CTE to facilitate client loading, a feature absent in other homologs and orthologs. We propose that the conserved motifs within the CTE are pivotal in mediating these interactions, although the precise mechanism remains to be elucidated. These findings raise intriguing questions about the evolutionary adaptation of the plastid HSP90C CTE and its functional significance, warranting further investigation.

### 4.2. The tSP Is Sufficient for the SecA1 Recruitment Process

The chloroplast SEC1 translocon is a relatively understudied protein translocation system that transports essential photosynthetic subunits into the thylakoid membrane. While the functional cycle of the chloroplast SEC1 translocon remains undefined, its structural similarities to bacterial Sec translocases suggest potential functional parallels [44]. SecA interacts with SecB and client proteins in bacterial systems to form a ternary complex in the cytoplasm [45]; however, whether this feature is conserved in plastid systems remains uncertain. Our findings demonstrate that the thylakoid signal peptide is critical for SEC1 substrate translocation. ATPase activity assays reveal distinct binding dynamics between SecA1 and its client protein compared with those between HSP90C and its client protein, suggesting unique binding modalities for each step of the translocation. These results have refined our understanding of the HSP90C-SecA1 interaction, leading to a refined and more detailed mechanistic model of their roles in thylakoid protein translocation (Figure 6). This revised model details the formation of a binary complex between the SEC client protein and HSP90C, in which HSP90C’s ATPase activity is significantly reduced, adopting a semi-inhibited conformation with the tSP exposed, thereby serving as a docking site for SecA1 recruitment. The recruitment of SecA1 monomers into the stromal compartment by the tSP specifically enhances SecA1’s ATPase activity, facilitating its subsequent targeting and translocation through the SecYE translocon.

Our investigation into the interaction between SecA1 and PsbO1 proteins highlights a clear preference for the tSP. Previous studies have demonstrated that bacterial signal peptides bind to SecA in liposomes and stimulate SecA-lipid ATPase activity [46,47], a phenomenon also observed in our data. By fitting the data to a classical Michaelis-Menten model, we determined the binding constants of PsbO1 to SecA1. The ATPase activity represented here is the basal activity of SecA1, unmodulated by auxiliary components such as glycolipids, cofactors, chaperones, or other SEC1 subunits. The relative stimulation of ATPase activity was exclusively attributed to the presence of the tSP. Collectively, these data indicate that structural elements of the tSP preferentially interact with SecA1, enhancing its activity. Stimulating SecA1’s basal ATPase activity induced by the tSP reflects a significant enhancement of its enzymatic activity. The basal, unbound conformation of SecA1 is likely not structurally optimized for translocation, resulting in a modest increase in ATPase activity. However, incorporating auxiliary factors such as lipids, metal ions, additional SEC subunits, and chaperones would likely substantially increase SecA1 activity [45,46,48]. The increases in the catalytic hydrolysis are more pronounced with the iPsbO1 compared with mPsbO1, suggesting that signal peptide binding biases SecA1 toward a conformation more favorable for translocation.

Previous studies have reported a 25–35% increase in SecA1 ATPase activity when synthetic tSP sequences analogous to pea plastocyanin and the 33-kDa subunit of the oxygen-evolving complex (OE33, a wheat PsbO1 analog) were compared to E. coli signal peptides from both SEC-dependent (alkaline phosphatase and β-lactamase) and TAT-dependent (SufI) pathways [41]. However, this ATPase stimulation was attributed to the presence of lipid mixtures (neutral galactolipid digalactosyldiacylglycerol and anionic dioleylphosphatidylglycerol in an 8:2 ratio) rather than the signal sequences alone. They do not show the stimulation caused by the signal sequence alone. It has been well characterized and reported that bacterial SecA adopts a distinct conformation, dissociates from dimers to monomers, and becomes ATPase-cycling competent upon association with acidic phospholipids such as phosphatidylglycerol (PG) [49,50,51,52]. The ATPase stimulation observed by Sun et al. in 2007 [41] is likely attributable to lipid-induced activation, inflating the reported values. In contrast, our study includes the mature domains for all PsbO1 constructs used, better reflecting the in vivo context. It excludes lipid-induced effects and focuses on the ATPase stimulation directly caused by the tSP of PsbO1. Our findings demonstrate that the tSP in SEC client proteins initiates SecA1 binding and stimulates its ATPase activity, thereby priming the ternary complex within the stroma and driving subsequent translocation.

### 4.3. The Subsequent Journey for the Ternary Complex

The work from our group has resolved that the interaction between HSP90C and SecA1 in the chloroplast stroma and hypothesized the formation of a ternary complex as a prelude to the thylakoid translocation process. BiFC analysis has previously demonstrated that the interaction between HSP90C and SecA1 in vivo depends on their respective ATPase activities [9]. Inhibiting HSP90C ATPase activity with GDA strongly reduced the interaction on the thylakoid membrane, while inhibiting SecA1 ATPase activity with sodium azide strongly reduced the stromal interaction. This suggests that the localization and stability of the interaction are ATPase-dependent, with active HSP90C being crucial for maintaining the interaction at the thylakoid membrane. In bacterial systems, SecB facilitates preprotein translocation by specifically interacting with membrane-bound SecA. This interaction exhibits varying affinities, with a low binding affinity in solution (dissociation constant ~1.6 µM) [23], which significantly increases when SecA is associated with the membrane-embedded SecYEG complex (10–30 nM) [24]. The affinity becomes even stronger (~10 nM) when SecB is loaded with a polypeptide ligand [53]. This shows the dynamic regulation of binding affinities during translocation. Whether this mechanism is conserved in chloroplast thylakoid systems remains an open question. Further insight into how SecA1 docks onto the cpSecYE channel and influences its binding affinity to HSP90C when loaded with an intermediate client pre-protein will provide critical mechanistic insights into the conservation of chloroplast protein translocation pathways with bacterial homologs.

Furthermore, in bacterial systems, the SecB binding site on SecA is primarily located at the extreme C-terminal tail (CTT) of SecA, especially towards the last 22 amino acids [24,54]. The CTT region of SecA doubles as a zinc-containing domain, which is essential for SecB interaction, and shows disruption of interaction through zinc chelation [55]. NMR studies suggest minimal structural changes in the SecA C-terminal region upon SecB binding, with the C-terminal tails of SecA projected beneath the SecA dimer [52,56]. The crystal structure of SecA reveals that NBD1, NBD2, PPXD, HSD, HWD, and CTT zinc-containing domains all facilitate interaction with the SecB–preprotein complex, positioning the preprotein near PPXD for efficient transfer [56,57]. Whether the CTT structure of chloroplast SecA1 also conserves the same functionality as that of bacterial SecA remains to be explored. Together, these investigations provide insights into the final stage of the HSP90C-assisted SEC1 translocation process.

## 5. Conclusions

In summary, this study utilized the photosystem II extrinsic protein PsbO1 as a model protein that is synthesized in the cytosol, imported into chloroplast stroma and then transported into the thylakoid, and delineated how the stromal chaperone HSP90C and the SEC translocation motor protein SecA bind, stabilize and guide it to the thylakoid lumen via the distinct binding regions. We identified both high- and low-affinity client-binding sites on the molecular chaperone, which are evolutionarily conserved. Together with the biochemical study of SecA1, we provided detailed mechanistic insight into why land plant chloroplast HSP90C CTE regions maintain highly conserved motifs such as DPW, and how some thylakoid lumen proteins may pass through the stroma and the SEC translocon with the aid of the HSP90 family molecular chaperone.

## Data Availability

The original contributions presented in this study are included in the article/Supplementary Material. Further inquiries can be directed to the corresponding author.

## Supplementary Materials

The following supporting information can be downloaded at https://www.mdpi.com/article/10.3390/biom16060903/s1: Table S1: Primers used for cloning and site-directed mutagenesis; Table S2: Plasmid constructs used for *E. coli* protein expression and yeast two-hybrid; Table S3: HSP90 sequence input for MD simulations and pairwise sequence alignment. Figure S1: Protein purification and quality analysis; Figure S2: Original Western blot images; Figure S3: *Arabidopsis thaliana* HSP90C structure predicted by AlphaFold3; Figure S4: AlphaFold3-predicted structural models of SecA1 and variants; Figure S5: AlphaFold3-predicted structural models of PsbO1 and variants.

## Abbreviations

The following abbreviations are used in this manuscript:

HSP90C	Chloroplast localized heat shock protein 90
SecA1	Chloroplast SEC translocase A1 subunit
PsbO1	Photosystem II subunit O isoform 1
*i*PsbO1	Intermediate stromal PsbO1 containing thylakoid signaling peptide
cTP	Chloroplast targeting peptide
tSP	Thylakoid signaling peptide
TAT	Twin-arginine translocation
CTE	C-terminal extension
HSD	SecA1 helical scaffolding domain
HWD	SecA1 helical wing domain
IRA1	SecA1 intramolecular Regulator of ATPase 1
NBD1/2	Nucleotide binding domain 1/2
PPXD	SecA1 preprotein crosslinking domain
IEM	Inner envelope membrane
OEM	Outer envelope membrane
MD	Molecular dynamics

## References

[1] Inoue H., Li M., Schnell D.J. (2013). An essential role for chloroplast heat shock protein 90 (Hsp90C) in protein import into chloroplasts. Proc. Natl. Acad. Sci. USA.

[2] Feng J., Fan P., Jiang P., Lv S., Chen X., Li Y. (2014). Chloroplast-targeted Hsp90 plays essential roles in plastid development and embryogenesis in *Arabidopsis* possibly linking with VIPP1. Physiol. Plant..

[3] Taipale M., Jarosz D.F., Lindquist S. (2010). HSP90 at the hub of protein homeostasis: Emerging mechanistic insights. Nat. Rev. Mol. Cell Biol..

[4] Jiang T., Mu B., Zhao R. (2020). Plastid chaperone HSP90C guides precursor proteins to the SEC translocase for thylakoid transport. J. Exp. Bot..

[5] Cao D., Froehlich J.E., Zhang H., Cheng C.L. (2003). The chlorate-resistant and photomorphogenesis-defective mutant cr88 encodes a chloroplast-targeted HSP90. Plant J..

[6] Wang X., Zhong J., Li B., Yang S., Wang P., Shao J., Meng J., Liu X., Yu F., Qi Y. (2025). AtHSP90.5 and AtFtsH12 synergistically regulate the accumulation of photosynthesis protein complexes and chloroplast development in *Arabidopsis*. Plant J..

[7] Oh S.E., Yeung C., Babaei-Rad R., Zhao R. (2014). Cosuppression of the chloroplast localized molecular chaperone HSP90.5 impairs plant development and chloroplast biogenesis in *Arabidopsis*. BMC Res. Notes.

[8] Schroda M., Muhlhaus T. (2009). A ‘foldosome’ in the chloroplast?. Plant Signal. Behav..

[9] Nair A.M., Jiang T., Mu B., Zhao R. (2024). Plastid molecular chaperone HSP90C interacts with the SecA1 subunit of Sec Translocase for thylakoid protein transport. Plants.

[10] Jiang T., Oh E.S., Bonea D., Zhao R. (2017). HSP90C interacts with PsbO1 and facilitates its thylakoid distribution from chloroplast stroma in *Arabidopsis*. PLoS ONE.

[11] Beckwith J. (2013). The Sec-dependent pathway. Res. Microbiol..

[12] Fernandez D.E. (2018). Two paths diverged in the stroma: Targeting to dual SEC translocase systems in chloroplasts. Photosynth. Res..

[13] Gold V.A., Duong F., Collinson I. (2007). Structure and function of the bacterial Sec translocon. Mol. Membr. Biol..

[14] Lang S., Pfeffer S., Lee P.H., Cavalie A., Helms V., Forster F., Zimmermann R. (2017). An Update on Sec61 Channel Functions, Mechanisms, and Related Diseases. Front. Physiol..

[15] Denks K., Vogt A., Sachelaru I., Petriman N.A., Kudva R., Koch H.G. (2014). The Sec translocon mediated protein transport in prokaryotes and eukaryotes. Mol. Membr. Biol..

[16] Lycklama A.N.J.A., Driessen A.J. (2012). The bacterial Sec-translocase: Structure and mechanism. Philos. Trans. R. Soc. Lond. B Biol. Sci..

[17] Linxweiler M., Schick B., Zimmermann R. (2017). Let’s talk about Secs: Sec61, Sec62 and Sec63 in signal transduction, oncology and personalized medicine. Signal Transduct. Target. Ther..

[18] Li Y., Singhal R., Taylor I.W., McMinn P.H., Chua X.Y., Cline K., Fernandez D.E. (2015). The Sec2 translocase of the chloroplast inner envelope contains a unique and dedicated SECE2 component. Plant J..

[19] Skalitzky C.A., Martin J.R., Harwood J.H., Beirne J.J., Adamczyk B.J., Heck G.R., Cline K., Fernandez D.E. (2011). Plastids contain a second sec translocase system with essential functions. Plant Physiol..

[20] Delepelaire P., Wandersman C. (1998). The SecB chaperone is involved in the secretion of the Serratia marcescens HasA protein through an ABC transporter. EMBO J..

[21] Lecker S., Lill R., Ziegelhoffer T., Georgopoulos C., Bassford P.J., Kumamoto C.A., Wickner W. (1989). Three pure chaperone proteins of *Escherichia coli*–SecB, trigger factor and GroEL–form soluble complexes with precursor proteins in vitro. EMBO J..

[22] Randall L.L., Topping T.B., Hardy S.J., Pavlov M.Y., Freistroffer D.V., Ehrenberg M. (1997). Binding of SecB to ribosome-bound polypeptides has the same characteristics as binding to full-length, denatured proteins. Proc. Natl. Acad. Sci. USA.

[23] den Blaauwen T., Terpetschnig E., Lakowicz J.R., Driessen A.J. (1997). Interaction of SecB with soluble SecA. FEBS Lett..

[24] Fekkes P., van der Does C., Driessen A.J. (1997). The molecular chaperone SecB is released from the carboxy-terminus of SecA during initiation of precursor protein translocation. EMBO J..

[25] Watanabe M., Blobel G. (1995). High-affinity binding of Escherichia coli SecB to the signal sequence region of a presecretory protein. Proc. Natl. Acad. Sci. USA.

[26] Klasek L., Inoue K., Theg S.M. (2020). Chloroplast Chaperonin-Mediated Targeting of a Thylakoid Membrane Protein. Plant Cell.

[27] Mu B., Nair A.M., Zhao R. (2024). Plastid HSP90C C-terminal extension region plays a regulatory role in chaperone activity and client binding. Plant J..

[28] Wei Y., Zhu B., Liu W., Cheng X., Lin D., He C., Shi H. (2021). Heat shock protein 90 co-chaperone modules fine-tune the antagonistic interaction between salicylic acid and auxin biosynthesis in cassava. Cell Rep..

[29] di Donato M., Geisler M. (2019). HSP90 and co-chaperones: A multitaskers’ view on plant hormone biology. FEBS Lett..

[30] Sahasrabudhe P., Rohrberg J., Biebl M.M., Rutz D.A., Buchner J. (2017). The Plasticity of the Hsp90 Co-chaperone System. Mol. Cell.

[31] Norby J.G. (1988). Coupled assay of Na+,K+-ATPase activity. Methods Enzymol..

[32] Abramson J., Adler J., Dunger J., Evans R., Green T., Pritzel A., Ronneberger O., Willmore L., Ballard A.J., Bambrick J. (2024). Accurate structure prediction of biomolecular interactions with AlphaFold 3. Nature.

[33] Bjelkmar P., Larsson P., Cuendet M.A., Hess B., Lindahl E. (2010). Implementation of the CHARMM Force Field in GROMACS: Analysis of Protein Stability Effects from Correction Maps, Virtual Interaction Sites, and Water Models. J. Chem. Theory Comput..

[34] MacKerell A.D., Banavali N., Foloppe N. (2000). Development and current status of the CHARMM force field for nucleic acids. Biopolymers.

[35] Huang J., MacKerell A.D. (2013). CHARMM36 all-atom additive protein force field: Validation based on comparison to NMR data. J. Comput. Chem..

[36] Grubmüller H., Heller H., Windemuth A., Schulten K. (1991). Generalized Verlet algorithm for efficient molecular dynamics simulations with long-range interactions. Mol. Simul..

[37] Di Pierro M., Elber R., Leimkuhler B. (2015). A Stochastic Algorithm for the Isobaric-Isothermal Ensemble with Ewald Summations for All Long Range Forces. J. Chem. Theory Comput..

[38] Darden T., York D., Pedersen L. (1993). Particle mesh Ewald: An N·log(N) method for Ewald sums in large systems. J. Chem. Phys..

[39] Hess B., Bekker H., Berendsen H.J., Fraaije J.G.E.M. (1997). LINCS: A linear constraint solver for molecular simulations. J. Comput. Biol..

[40] Wang R.Y., Noddings C.M., Kirschke E., Myasnikov A.G., Johnson J.L., Agard D.A. (2022). Structure of Hsp90-Hsp70-Hop-GR reveals the Hsp90 client-loading mechanism. Nature.

[41] Sun C., Rusch S.L., Kim J., Kendall D.A. (2007). Chloroplast SecA and Escherichia coli SecA have distinct lipid and signal peptide preferences. J. Bacteriol..

[42] Gelis I., Bonvin A.M., Keramisanou D., Koukaki M., Gouridis G., Karamanou S., Economou A., Kalodimos C.G. (2007). Structural basis for signal-sequence recognition by the translocase motor SecA as determined by NMR. Cell.

[43] Rutz D.A., Luo Q., Freiburger L., Madl T., Kaila V.R.I., Sattler M., Buchner J. (2018). A switch point in the molecular chaperone Hsp90 responding to client interaction. Nat. Commun..

[44] Haward S.R., Napier J.A., Gray J.C. (1997). Chloroplast SecA functions as a membrane-associated component of the Sec-like protein translocase of pea chloroplasts. Eur. J. Biochem..

[45] Cranford-Smith T., Huber D. (2018). The way is the goal: How SecA transports proteins across the cytoplasmic membrane in bacteria. FEMS Microbiol. Lett..

[46] Hsieh Y.H., Huang Y.J., Zhang H., Liu Q., Lu Y., Yang H., Houghton J., Jiang C., Sui S.F., Tai P.C. (2017). Dissecting structures and functions of SecA-only protein-conducting channels: ATPase, pore structure, ion channel activity, protein translocation, and interaction with SecYEG/SecDF*YajC. PLoS ONE.

[47] Wang L., Miller A., Kendall D.A. (2000). Signal peptide determinants of SecA binding and stimulation of ATPase activity. J. Biol. Chem..

[48] Jamshad M., Knowles T.J., White S.A., Ward D.G., Mohammed F., Rahman K.F., Wynne M., Hughes G.W., Kramer G., Bukau B. (2019). The C-terminal tail of the bacterial translocation ATPase SecA modulates its activity. Elife.

[49] Ahn T., Kim H. (1998). Effects of nonlamellar-prone lipids on the ATPase activity of SecA bound to model membranes. J. Biol. Chem..

[50] Dalal K., Chan C.S., Sligar S.G., Duong F. (2012). Two copies of the SecY channel and acidic lipids are necessary to activate the SecA translocation ATPase. Proc. Natl. Acad. Sci. USA.

[51] Lill R., Cunningham K., Brundage L.A., Ito K., Oliver D., Wickner W. (1989). SecA protein hydrolyzes ATP and is an essential component of the protein translocation ATPase of Escherichia coli. EMBO J..

[52] Osborne A.R., Clemons W.M., Rapoport T.A. (2004). A large conformational change of the translocation ATPase SecA. Proc. Natl. Acad. Sci. USA.

[53] Hartl F.U., Lecker S., Schiebel E., Hendrick J.P., Wickner W. (1990). The binding cascade of SecB to SecA to SecY/E mediates preprotein targeting to the *E. coli* plasma membrane. Cell.

[54] Woodbury R.L., Hardy S.J., Randall L.L. (2002). Complex behavior in solution of homodimeric SecA. Protein Sci..

[55] Fekkes P., de Wit J.G., Boorsma A., Friesen R.H., Driessen A.J. (1999). Zinc stabilizes the SecB binding site of SecA. Biochemistry.

[56] Hunt J.F., Weinkauf S., Henry L., Fak J.J., McNicholas P., Oliver D.B., Deisenhofer J. (2002). Nucleotide control of interdomain interactions in the conformational reaction cycle of SecA. Science.

[57] Kimura E., Akita M., Matsuyama S., Mizushima S. (1991). Determination of a region in SecA that interacts with presecretory proteins in Escherichia coli. J. Biol. Chem..

