# Supplementary material for: Arabidopsis HSP90C and SecA1 Have Distinct Client-Binding Modalities to the Thylakoid SEC Client Protein PsbO1
Source: Biomolecules. 2026 Jun 18;16(6):903. doi: 10.3390/biom16060903 (PMC13296758; doi:10.3390/biom16060903)
Supplement: Supplementary file 1 [file biomolecules-16-00903-s001.zip › Figure S5_PsbO1 predicted and experiimentally solved structures.pdf]

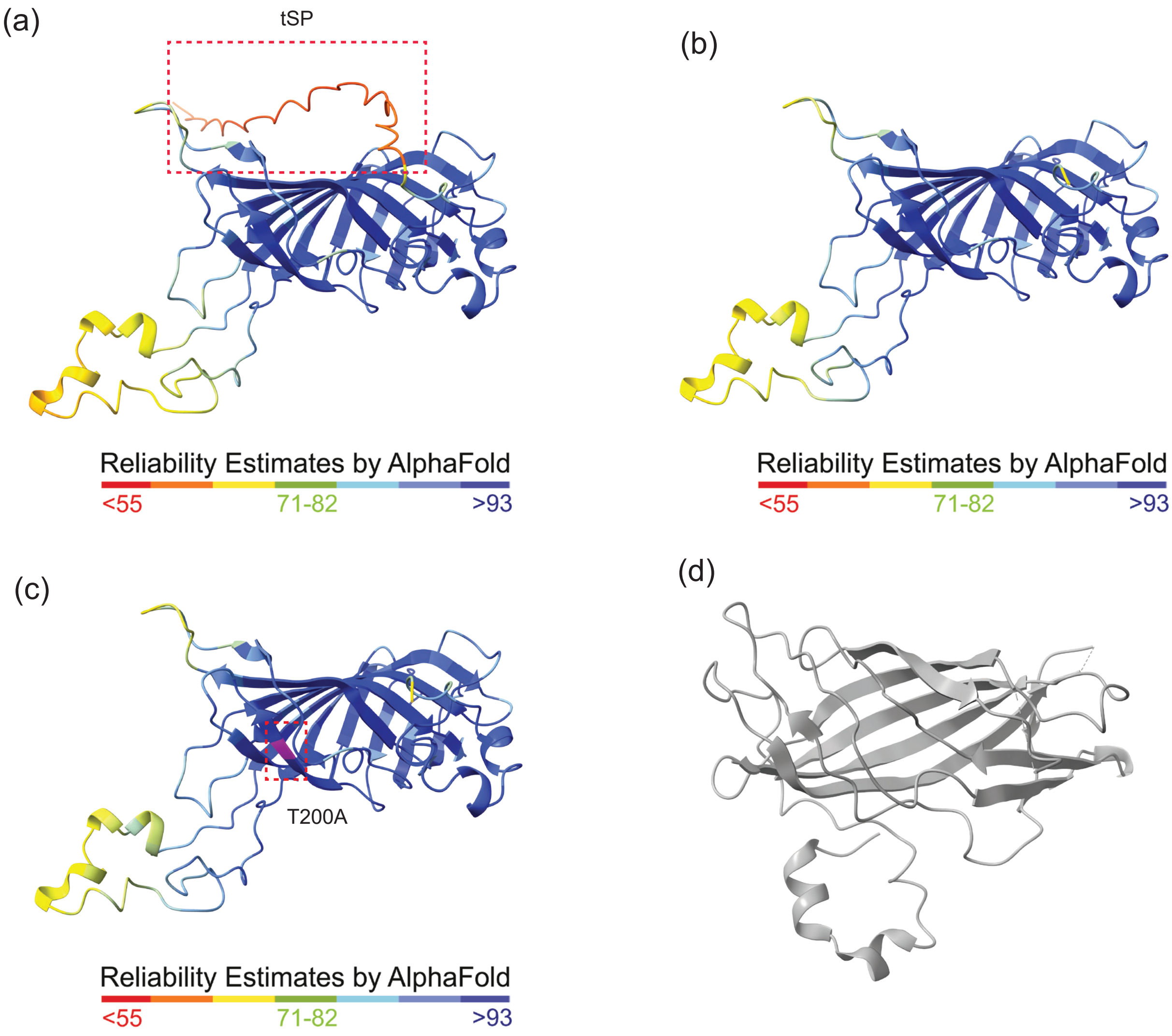

Figure S5. AlphaFold3-predicted structural models of PsbO1 and variants.
(a) Intermediate precursor PsbO1 (*i*PsbO1), encompassing the mature form and the thylakoid signal peptide (tSP; residues 59–332).
(b) Mature PsbO1 (*m*PsbO1), representing the processed form lacking the tSP (residues 85–332).
(c) Mature PsbO1 T200A point mutant (mPsbO1^{T200A}), identical to *m*PsbO1 but carrying a threonine-to-alanine substitution at residue 200, introduced in silico prior to simulation. The point mutation is highlighted in purple at position 200.
(d) Experimentally resolved PsbO1 chain extracted from the cryo-EM structure of th frame of reference. All predicted structures in panels (a–c) were generated using AlphaFold3 and (d) is PSII supercomplex from *Arabidopsis thaliana* (PDB: 5MDX), shown in grey as a structural frame of reference. All predicted structures in panels (a-c) were generated using AlphaFold3 and colored according to per residue predicted local distance difference test (pLDDT). Confidence scores: dark blue (pLDDT > 93, very high confidence), teal/-cyan (pLDDT: 71–82, confident), yellow (moderate), and red/orange (pLDDT < 55, low confidence) as indicated by the reliability scale bar. The experimentally resolved chain in panel (d) is shown in grey for reference. Structure visualization was performed in ChimeraX.
