# Supplementary material for: Arabidopsis HSP90C and SecA1 Have Distinct Client-Binding Modalities to the Thylakoid SEC Client Protein PsbO1
Source: Biomolecules. 2026 Jun 18;16(6):903. doi: 10.3390/biom16060903 (PMC13296758; doi:10.3390/biom16060903)

Original Western blot images

Original Western blot chemiluminescent image for Figure 1d Top.

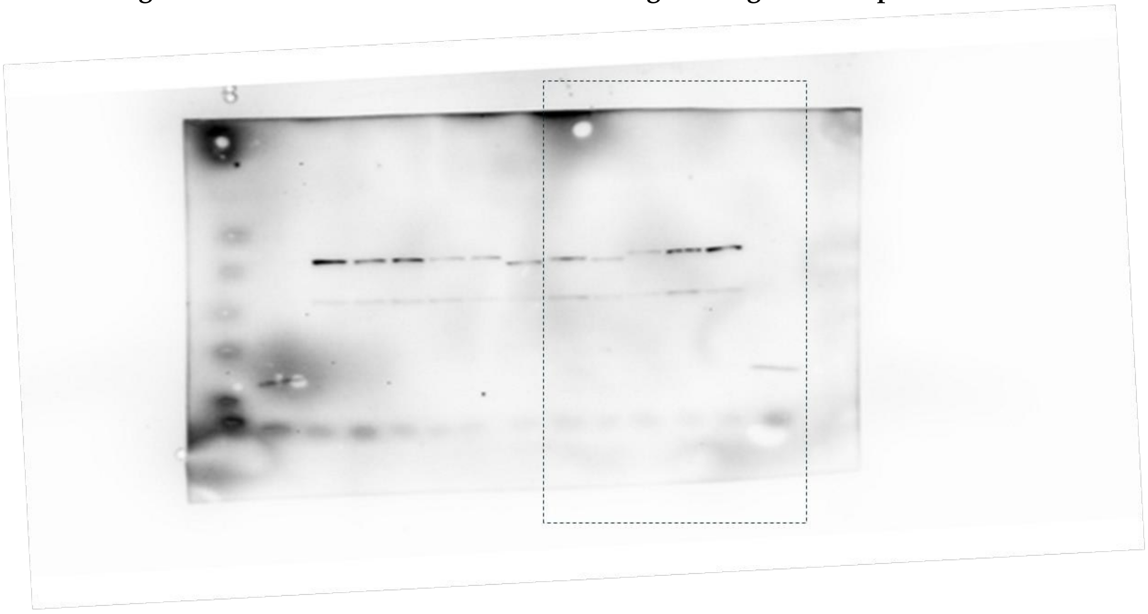

Original Western blot chemiluminescent image for Figure 1d middle.

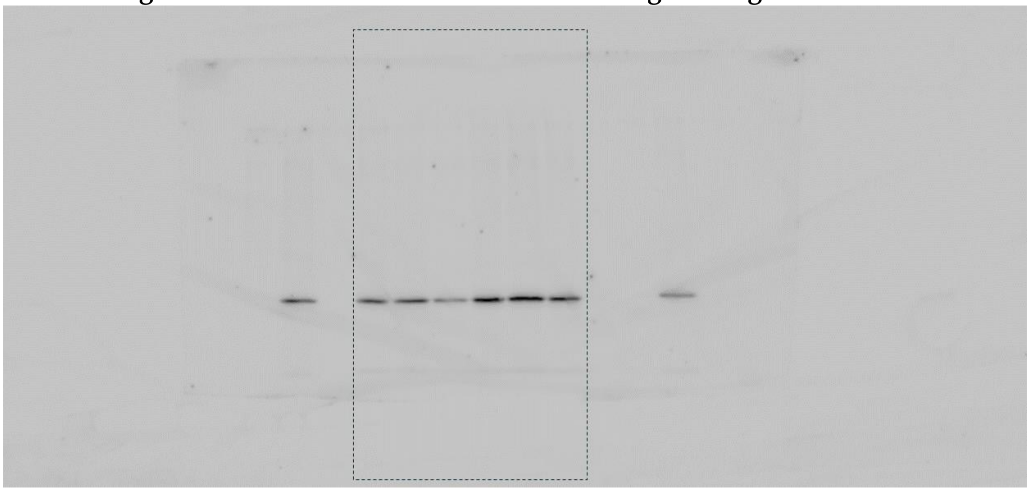

Original Western blot chemiluminescent image for Figure 1d bottom.

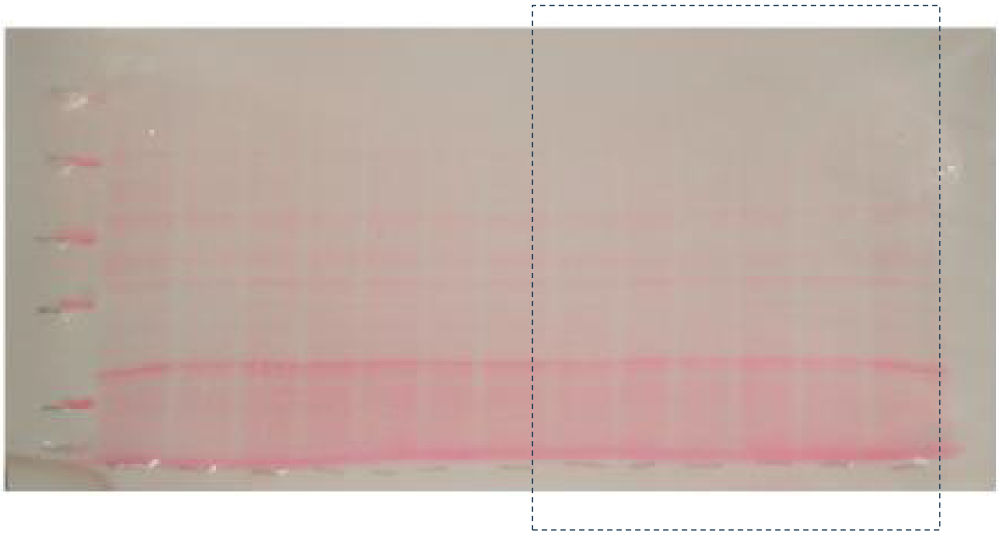

Supplement: Supplementary file 1 [file biomolecules-16-00903-s001.zip › Figure S2_Original_blot_images.pdf]
