# Supplementary material for: Arabidopsis HSP90C and SecA1 Have Distinct Client-Binding Modalities to the Thylakoid SEC Client Protein PsbO1
Source: Biomolecules. 2026 Jun 18;16(6):903. doi: 10.3390/biom16060903 (PMC13296758; doi:10.3390/biom16060903)
Supplement: Supplementary file 1 [file biomolecules-16-00903-s001.zip › Figure S1_Purification of PsbO1 and HSP90C variant proteins.pdf]

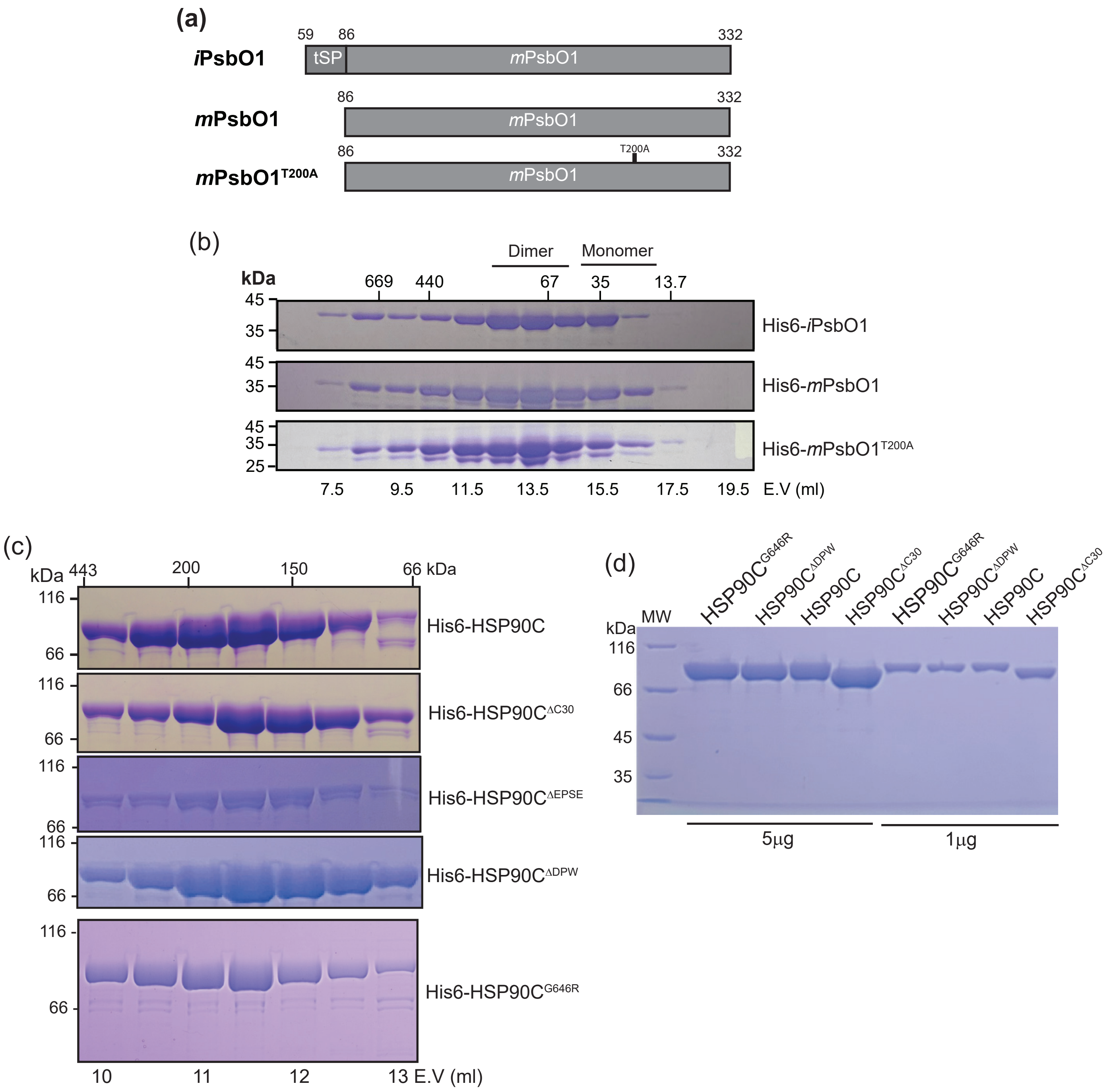

Figure S1. Protein purification and quality analysis.

(a) Schematic diagrams of *i*PsbO1, *m*PsbO1 and *m*PsbO1^{T200A}.

(b) SDS-PAGE analysis of purified His6-tagged *i*PsbO1, *m*PsbO1 and *m*PsbO1^{T200A}.

(c) Wild-type HSP90C as well as mutant HSP90Cs including those lacking last 30 amino acids (HSP90CΔC30), delection of EPSE (HSP90CΔEPSE), deletion of DPW (HSP90CΔDPW) motifs, and the one with glycine 646 mutated to arginine (HSP90C$^{G646R)}$ were purified by Ni-NTA and then separated by size exclusion chromatography analysis on Supdex 200 before subjected to 10% SDS-PAGE analysis. The estimated molecular weights from the column Superdex 200 and the elution volumes are indicated in the top and bottom, respectively.

(d) The final purified proteins His6-tagged HSP90C, HSP90C^{G646R}, HSP90C$^{\Delta DPW}$ and HSP90C$^{\Delta C30}$ are shown as examples and loaded with 5μg and 1μg, respectively. Gels were stained with Coomassie Brilliant Blue in (b, c, d).
