# Supplementary material for: Arabidopsis HSP90C and SecA1 Have Distinct Client-Binding Modalities to the Thylakoid SEC Client Protein PsbO1
Source: Biomolecules. 2026 Jun 18;16(6):903. doi: 10.3390/biom16060903 (PMC13296758; doi:10.3390/biom16060903)
Supplement: Supplementary file 1 [file biomolecules-16-00903-s001.zip › Figure_S3_HSP90C_pLDDT_cartoon_surface_overlap.pdf]

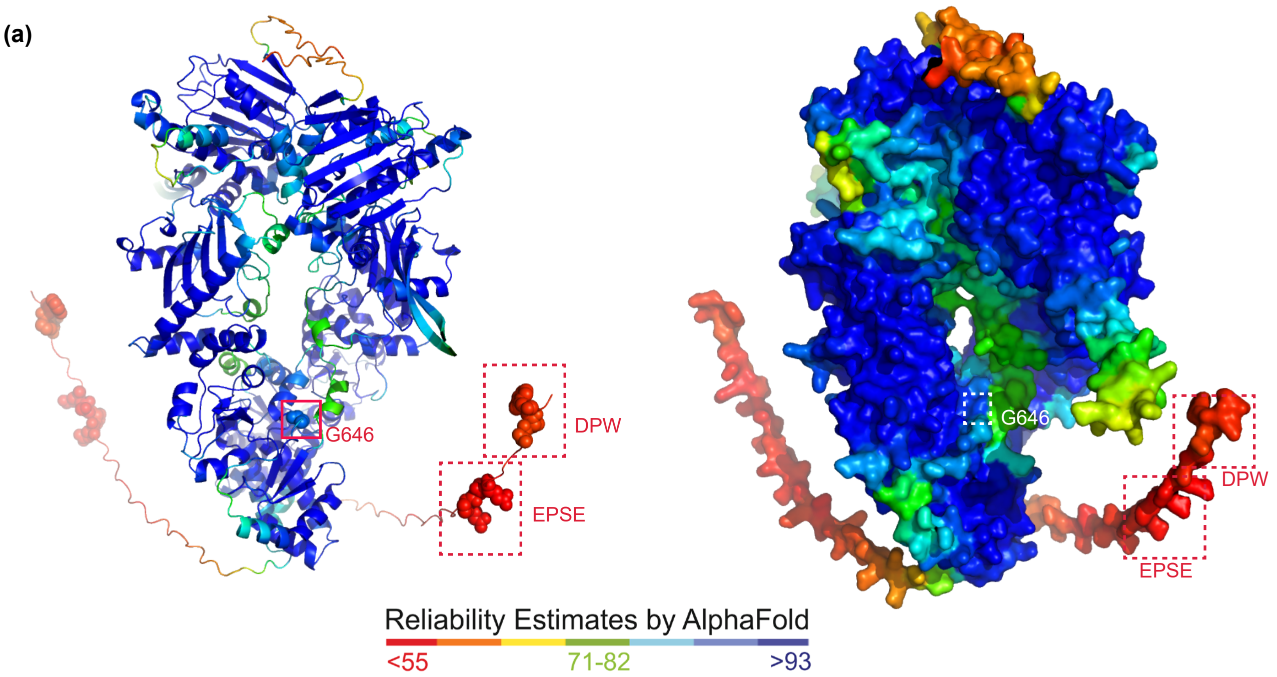

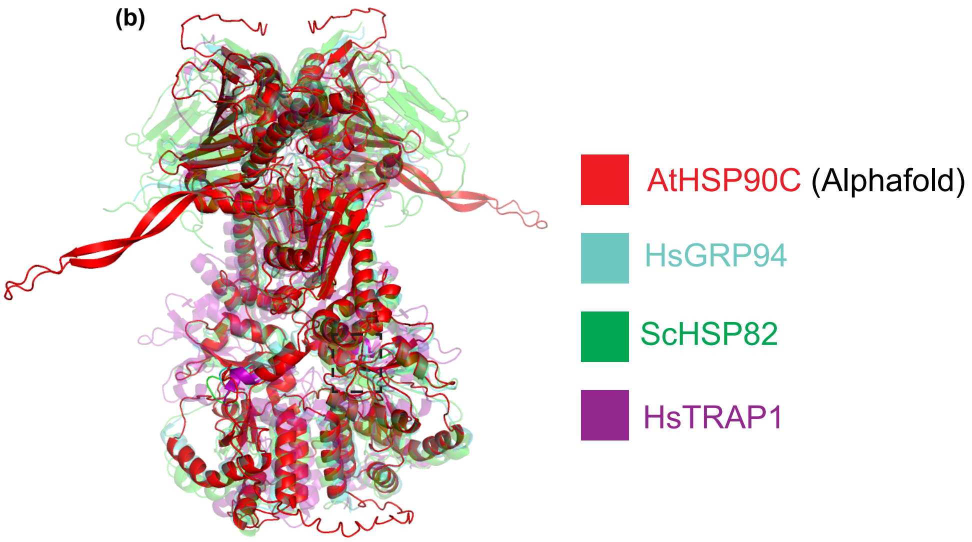

Figure S3 *Arabidopsis thaliana* HSP90C structure predicted by AlphaFold3.
(a) The predicted HSP90C from *Arabidopsis* (AtHSP90C) structure in ribbon cartoon (left) and the surface (right) model that are colored based on the reliabilty of predicted local distance difference test (pLDDT). The DPW, EPSE and G646 are shown in sphere models and highlighted within square boxes.
(b) AtHSP90C predicted structure was superimposed with experimentally solved homologous HSP90 family proteins from human (PDB: 8EOB), *Saccharomyces cerevisiae* (PDB:2CG9), and human mito-chondrial TRAP1 (PDB:7KCK).
