# Supplementary material for: Arabidopsis HSP90C and SecA1 Have Distinct Client-Binding Modalities to the Thylakoid SEC Client Protein PsbO1
Source: Biomolecules. 2026 Jun 18;16(6):903. doi: 10.3390/biom16060903 (PMC13296758; doi:10.3390/biom16060903)
Supplement: Supplementary file 1 [file biomolecules-16-00903-s001.zip › Figure S4_Predicted_Arabidopsis SecA1 and solved homologue structure..pdf]

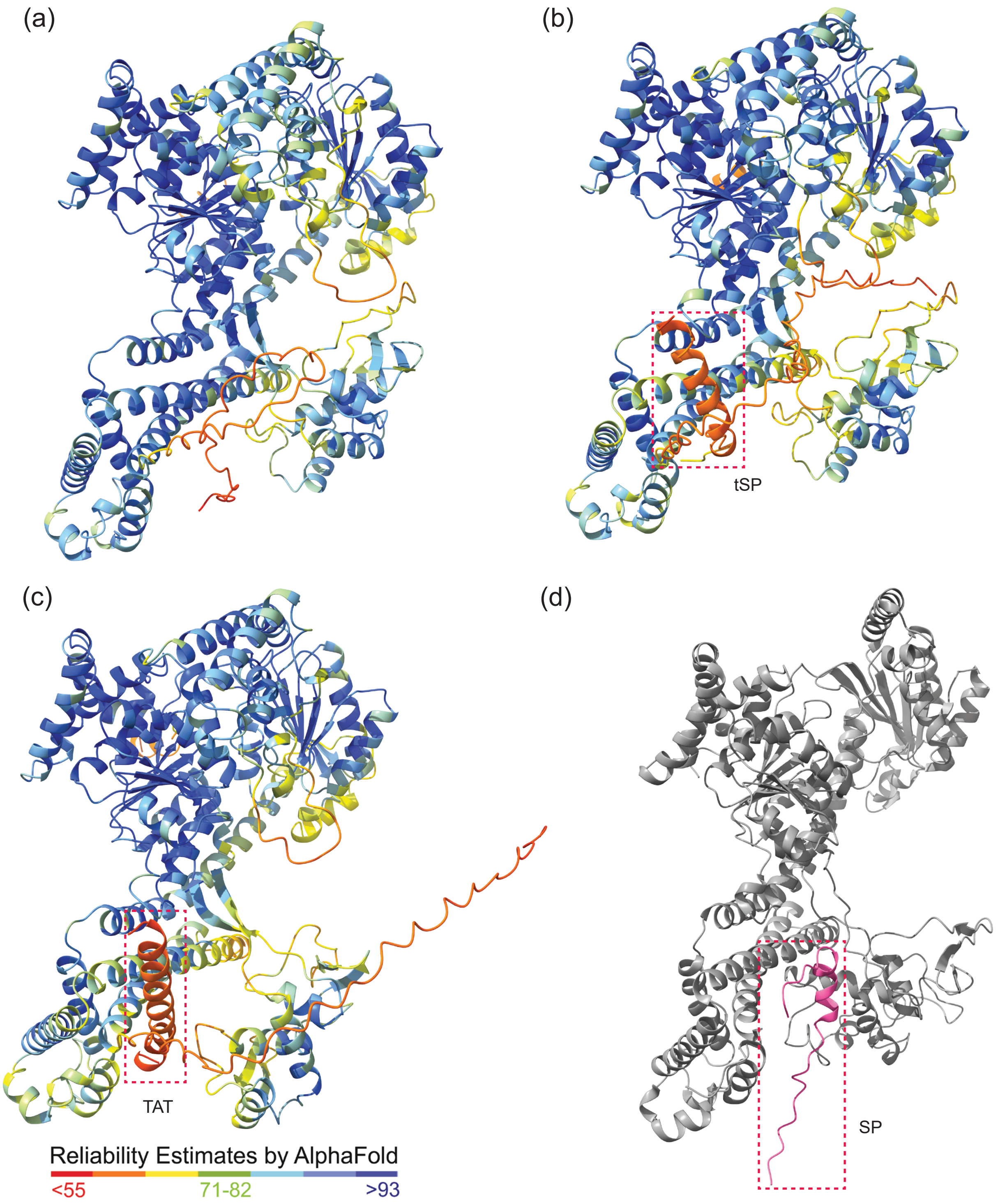

Figure S4. AlphaFold3-predicted structural models of SecA1 and variants with rexperimentally resolved bacterial SecA structure.

(a-c) Predicted mature *Arabidopsis* SecA1 (*m*SecA1) alone (a) and the *m*SecA1 in complex with the thylakoid signal peptide (tSP; residues 59–85) derived from *Arabidopsis* PsbO1 (b), or with the twin-arginine translocation (TAT) signal sequence (residues 45–75) derived from *Arabidopsis* PsbQ1. The colors in panels (a-c) represent per-residue predicted local distance difference test (pLDDT) confidence scores and the reliability scale bar is shown in panels (c) only. The tSP or TAT signal sequences were highlighted in dashed line rectangle boxes.

(d) Experimentally resolved bacterial SecA from *Escherichia coli* (PDB: 2VDA) shown with its native signal peptide (maltoporin signal sequence; residues 1–27) as a structural frame of reference for domain architecture comparison. The experimentally resolved structure in panel (d) is shown in grey for reference with the signal peptide maltoporin chain distinguished in pink and within dashed pink rectangle box. Structure visualization were performed using ChimeraX.
